# Rezafungin Versus Caspofungin in a Phase 2, Randomized, Double-blind Study for the Treatment of Candidemia and Invasive Candidiasis: The STRIVE Trial

**DOI:** 10.1093/cid/ciaa1380

**Published:** 2020-09-21

**Authors:** George R Thompson, Alex Soriano, Athanasios Skoutelis, Jose A Vazquez, Patrick M Honore, Juan P Horcajada, Herbert Spapen, Matteo Bassetti, Luis Ostrosky-Zeichner, Anita F Das, Rolando M Viani, Taylor Sandison, Peter G Pappas

**Affiliations:** 1 Department of Internal Medicine Division of Infectious Diseases and Department of Medical Microbiology and Immunology, University of California Davis Medical Center, Sacramento, California, USA; 2 Department of Infectious Diseases, Hospital Clínic de Barcelona, IDIBAPS, University of Barcelona, Spain; 3 Department of Medicine and Infectious Diseases, Evangelismos General Hospital, Athens, Greece; 4 Department of Medicine/Division of Infectious Disease, Medical College of Georgia/Augusta University, Augusta, Georgia, USA; 5 Department of Intensive Care, Brugmann University Hospital, Brussels, Belgium; 6 Department of Infectious Diseases, Hospital del Mar, Institut Hospital del Mar d’Investigacions Mèdiques (IMIM), Universitat Autònoma de Barcelona (UAB), Universitat Pompeu Fabra (UPF), Barcelona, Spain; 7 Department of Intensive Care, University Hospital Brussels, Brussels, Belgium; 8 Department of Health Sciences University of Genoa and Policlinico San Martino IST, Genoa, Italy; 9 Division of Infectious Diseases, McGovern Medical School, Houston, Texas, USA; 10 AD Stat Consulting, Guerneville, California, USA; 11 Cidara Therapeutics, San Diego, California, USA; 12 Department of Internal Medicine Division of Infectious Diseases, University of Alabama at Birmingham, Birmingham, Alabama, USA

**Keywords:** echinocandins, rezafungin, candidemia, systemic antifungal therapy

## Abstract

**Background:**

Rezafungin (RZF) is a novel echinocandin exhibiting distinctive pharmacokinetics/pharmacodynamics. STRIVE was a phase 2, double-blind, randomized trial designed to compare the safety and efficacy of RZF once weekly (QWk) to caspofungin (CAS) once daily for treatment of candidemia and/or invasive candidiasis (IC).

**Methods:**

Adults with systemic signs and mycological confirmation of candidemia and/or IC were randomized to RZF 400 mg QWk (400 mg), RZF 400 mg on week 1 then 200 mg QWk (400/200 mg), or CAS 70 mg as a loading dose followed by 50 mg daily for ≤4 weeks. Efficacy assessments included overall cure (resolution of signs of candidemia/IC + mycological eradication) at day 14 (primary endpoint), investigator-assessed clinical response at day 14, and 30-day all-cause mortality (ACM) (secondary endpoints), and time to negative blood culture. Safety was evaluated by adverse events and ACM through follow-up.

**Results:**

Of 207 patients enrolled, 183 were in the microbiological intent-to-treat population (~21% IC). Overall cure rates were 60.5% (46/76) for RZF 400 mg, 76.1% (35/46) for RZF 400/200 mg, and 67.2% (41/61) for CAS; investigator-assessed clinical cure rates were 69.7% (53/76), 80.4% (37/46), and 70.5% (43/61), respectively. In total, 30-day ACM was 15.8% for RZF 400 mg, 4.4% for RZF 400/200 mg, and 13.1% for CAS. Candidemia was cleared in 19.5 and 22.8 hours in RZF and CAS patients, respectively. No concerning safety trends were observed; ACM through follow-up was 15.2% (21/138) for RZF and 18.8% (13/69) for CAS.

**Conclusions:**

RZF was safe and efficacious in the treatment of candidemia and/or IC.

**Clinical Trials Registration:**

NCT02734862

## Teaser

In a phase 2, randomized, double-blind, multicenter clinical trial conducted to evaluate rezafungin compared with caspofungin followed by fluconazole for primary treatment of patients with proven candidemia or invasive candidiasis, rezafungin demonstrated favorable safety and efficacy.

Invasive candidiasis (IC) remains a significant cause of morbidity and mortality [1]. Despite significant advances in antifungal therapy over the past 2 decades, mortality rates remain between 30% and 60% [2, 3]. Echinocandins are recommended first-line agents in the treatment of most types of IC [1, 4]. Patients treated with an echinocandin show significantly better survival rates than patients treated with other classes of antifungals; however, treatment failure with echinocandin therapy occurs in approximately 40% of cases [2] and presents an opportunity for improvement. The efficacy of echinocandins is reliant on concentration-dependent effects and drug levels within target tissue sites. Currently available echinocandins and dosing strategies may be insufficient to eradicate *Candida* spp. or to prevent the development of resistance [5–10].

Rezafungin (RZF) is a next-generation echinocandin with significant pharmacokinetic advantages, including a prolonged half-life (~133 hours) and high plasma drug concentrations early in the course of therapy, allowing for front-loaded, extended-interval dosing. Dose-proportional pharmacokinetics have demonstrated minimal interpatient variability and a favorable safety profile [11]. Preclinical studies have established the efficacy of RZF in animal models of candidemia, IC, and *Candida* biofilms [5, 9, 12–15]. In addition, phase 1 studies have demonstrated the safety and tolerability of RZF in healthy volunteers [11, 16]. Here we present the results of STRIVE, a phase 2 trial designed to evaluate the safety and efficacy of intravenous (IV) RZF, compared with caspofungin (CAS) and optional oral fluconazole stepdown therapy, in the treatment of candidemia/IC.

## METHODS

### Study Design

STRIVE (Clinicaltrials.gov, NCT02734862) was a phase 2, randomized, double-blind, double-dummy, multicenter trial conducted in 44 centers and in 10 countries (Supplementary Table 1) in accordance with current country and local regulations, the International Conference on Harmonisation Good Clinical Practice, and the Declaration of Helsinki. Ethics committees or institutional review boards at participating sites approved the protocol and all amendments. All patients provided written informed consent.

### Patient Disposition

Eligible patients (male and female; ages ≥18 years) had systemic signs of infection and mycological evidence of candidemia and/or IC from a sample within 96 hours prior to randomization. Key exclusion criteria were certain forms of IC (ie, septic arthritis in a prosthetic joint, osteomyelitis, endocarditis, or myocarditis); *Candida* infections of the eye (eg, endophthalmitis) or the central nervous system; neutropenia (absolute neutrophil count ≤500/µL); alanine aminotransferase or aspartate aminotransferase levels >10-fold the upper limit of normal or severe hepatic impairment with a history of chronic cirrhosis (Child-Pugh score >9); and >48 hours of prior systemic antifungal therapy.

### Randomization, Stratification, and Blinding

The trial consisted of 2 parts with different randomization schedules, each using block randomization, and stratified based on candidemia versus IC. Patients in part A were randomized (1:1:1) to receive RZF IV once weekly for 2–4 weeks at either 400 mg (group 1) or 400 mg on week 1 followed by 200 mg on subsequent weeks (group 2), or CAS once daily (70 mg loading dose followed by 50 mg daily with an optional oral stepdown available after day 3) (group 3). In part B, patients were randomized (2:1) to receive RZF IV once weekly or CAS once daily. The initial RZF dosing regimen was 400 mg once weekly (identical to group 1 of part A). After review of unblinded part A data, the RZF regimen was modified to 400 mg on week 1 followed by 200 mg weekly thereafter (identical to group 2 of part A) to align with the dosing regimen selected for phase 3. Patients in part B who were randomized to 400 mg once weekly remained on that regimen through treatment completion. All patients and study personnel interacting with patients were blinded to treatment assignment.

RZF IV was administered on days 1 and 8, with an optional dose on day 15 as determined by the Investigator. Patients with IC may have received an additional optional dose on day 22. CAS was administered once daily, up to 21 days for candidemia and up to 28 days for IC with or without candidemia. After at least 3 days on IV therapy, patients who met qualifying criteria could be switched to the oral stepdown regimen. In the CAS group, the oral stepdown regimen was fluconazole 800 mg on the first day of the switch, followed by 400 mg/day and weekly placebo IV; in the RZF groups, the regimen comprised oral placebo capsules and weekly RZF IV. Patients continued to receive placebo, IV or oral, as needed to preserve blinding through treatment completion.

### Efficacy and Safety Assessments

All consented and randomized patients were included in the intent-to-treat (ITT) population. All patients who received any amount of study drug were included in the safety population, which was used to assess safety outcomes. Patients in the safety population who had documented *Candida* infection at baseline were included in the microbiological ITT (mITT) population used to assess primary and secondary efficacy outcomes.

The primary efficacy outcome was overall response (with overall cure defined as resolution of clinical signs of candidemia/IC + mycological eradication) at day 14 (±1 day). Mycological response was defined as success (mycological eradication, presumed or documented, with no change in antifungal therapy), failure (mycological persistence, presumed or documented), or indeterminate (culture specimen or result not available or patient lost to follow-up). Mycological eradication was documented by 2 sequential negative blood cultures ≥12 hours apart for patients with candidemia or by negative results on the most recent culture before day 14 for patients with a qualifying positive culture from a normally sterile site. Mycological eradication was presumed if follow-up culture was not available in a patient with clinical cure and with, in cases of IC, resolution/improvement of IC-related baseline radiographic abnormalities. Secondary efficacy outcomes included overall, mycological, and Investigator-assessed clinical response at day 5 (overall and mycological responses only), day 14 (±1 day), day 28 (±2 days; for IC only), and follow-up (days 45–52 for candidemia only or days 52–59 for IC, with or without candidemia), as well as all-cause mortality (ACM) at day 30.

Safety was determined by adverse events (AEs), treatment-emergent AEs (TEAEs), ACM through follow-up, and vital signs, laboratory and EKG testing.

### Statistical Analysis

The trial was not powered for inferential analysis. A sufficient number of patients were randomized in part A for an initial substantive analysis of safety and tolerability and an initial estimate of efficacy. In part A, assuming a 73% overall cure rate, the sample size of 30 patients in each of the RZF groups would yield a 95% confidence interval (CI) of 53.8% to 87.5%. With the addition of part B patients, again assuming a 73% overall cure rate, a total approximate sample size of 60 patients in the overall RZF group would yield a 95% CI of 60.0% to 83.7%. Analyses were performed using SAS version 9.3. Continuous and categorical data were summarized descriptively. Exact 2-sided 95% CIs for the point estimates of overall cure and clinical cure were determined using the Clopper-Pearson method. ACM and time to negative blood culture were analyzed using Kaplan-Meier methods. For time to negative blood cultures, the first of the 2 required negative cultures was used as the time of culture clearance, and a log-rank test (post hoc analysis) was used to determine differences between treatment groups.

## RESULTS

### Study Conduct and Patient Disposition

**Figure 1.**
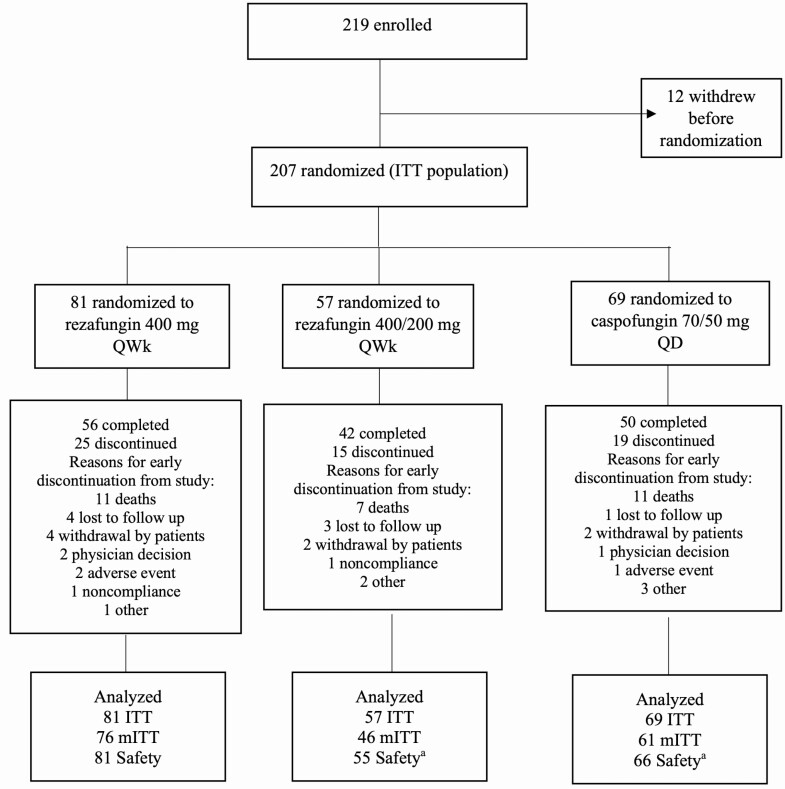
Patient flow. ITT population included all consented and randomized patients. Safety population included all patients who received any amount of study drug. The mITT population included all patients in the safety population with documented *Candida* infection. *Two patients who were randomized to Group 2 received caspofungin and were included in safety analyses of Group 3. Abbreviations: ITT, intent-to-treat; MITT, microbiological intent-to-treat; OD, once daily; QWk, once weekly.

Between July 2016 and April 2019, 219 patients were screened and 207 were randomized (ITT population); 202 (97.6%) received at least 1 dose of study drug (safety population). A total of 183 patients (88.4%) in the safety population had documented *Candida* infection and comprised the mITT population (Figure 1). Treatment groups were well balanced and matched in demographics and baseline characteristics (Table 1, Supplementary Table 2). The median duration of IV and oral treatment combined in the safety population was 14.0 days (range, 1–28 days) in each of the 3 treatment groups. The median duration of IV treatment was 14.0 days in both RZF groups (range, 1–28 days [400 mg], 1–22 days [400/200 mg]) and 12.0 days (1–28 days) in the CAS group. In the CAS group, 24 of 68 patients (35.3%) were switched to oral stepdown for a median duration of 9.0 days (range, 2–18 days). The timing of switch is summarized in Supplementary Table 3. Of patients with candidemia who had a central venous catheter present at screening, similar proportions across treatment groups underwent catheter removal within 48 hours after diagnosis of candidemia (33.3%, 17/51 [RZF 400 mg]; 35.7%, 10/28 [RZF 400/200 mg group]; and 38.6%, 17/44 [CAS]). The types of central venous catheters that were present in each treatment arm are shown in Supplementary Table 4.

**Table 1.** Demographics and Baseline Characteristics (Intent-to-Treat [ITT] Population)—Parts A and B Combined

Demographic or Characteristic	Rezafungin Once Weekly 400 mg N = 81	Rezafungin Once Weekly 400 mg/200 mg N = 57	Caspofungin Once Daily 70 mg/50 mg N = 69
Age in years			
Mean ± SD	59 ± 16	60 ± 16	59 ± 16
Range	24–88	24–91	24–93
<65 years, n (%)	49 (60.5)	32 (56.1)	40 (58.0)
≥65 years, n (%)	32 (39.5)	25 (43.9)	29 (42.0)
Sex, n (%)			
Male	44 (54.3)	36 (63.2)	38 (55.1)
Race, n (%)			
Asian	0	1 (1.8)	3 (4.3)
Black or African American	8 (9.9)	7 (12.3)	4 (5.8)
White	69 (85.2)	44 (77.2)	59 (85.5)
Other	4 (4.9)	2 (3.5)	0
Not reported	0	3 (5.3)	3 (4.3)
Ethnicity, n (%)			
Hispanic/Latino	8 (9.9)	9 (15.8)	7 (10.1)
Not Hispanic/Latino	73 (90.1)	46 (80.7)	59 (85.5)
Not reported	0	2 (3.5)	3 (4.3)
Diagnosis, n (%)			
Candidemia	62 (76.5)	46 (80.7)	56 (81.2)
Invasive candidiasis	19 (23.5)	11 (19.3)	13 (18.8)
BMI^a^, mean ± SD kg/m^2^	26.9 ± 7.17	26.8 ± 8.57	26.6 ± 5.63
APACHE II Category, n (%)			
0–9	23 (28.4)	15 (26.3)	17 (24.6)
10–19	39 (48.1)	26 (45.6)	37 (53.6)
≥20	17 (21.0)	14 (24.6)	9 (13.0)
Not available	2 (2.5)	2 (3.5)	6 (8.7)
APACHE II Score	n = 79	n = 55	n = 63
Mean ± SD	13.4 ± 7.13	14.1 ± 6.72	14.0 ± 7.39
Range	2.0–31.0	2.0–28.0	1.0–35.0

Abbreviations: APACHE II, acute physiology and chronic health evaluation II; BMI, body mass index; SD, standard deviation.

^a^BMI calculated by dividing weight (kg) by height (m^2^) based on patients with available data.

### Distribution of *Candida* Species

The *Candida* species isolated at baseline were predominantly *Candida albicans* (49.7%), followed by *Candida glabrata* (20.2%), *Candida parapsilosis* (15.3%), and *Candida tropicalis* (12.0%) (Table 2).

**Table 2.** Fungal Pathogens at Baseline (Microbiological Intent-to-Treat [mITT] Population)—Parts A and B Combined

*Candida* Species, n (%)^a^	Rezafungin Once Weekly 400 mg N = 76	Rezafungin Once Weekly 400 mg/200 mg N = 46	Caspofungin Once Daily 70 mg/50 mg N = 61	Total N = 183
*C. albicans*	38 (50.0)	19 (41.3)	34 (55.7)	91 (49.7)
*C. glabrata*	13 (17.1)	14 (30.4)	10 (16.4)	37 (20.2)
*C. parapsilosis*	10 (13.2)	7 (15.2)	11 (18.0)	28 (15.3)
*C. tropicalis*	9 (11.8)	7 (15.2)	6 (9.8)	22 (12.0)
*C. krusei*	1 (1.3)	3 (6.5)	1 (1.6)	5 (2.7)
*C. dubliniensis*	4 (5.3)	0	1 (1.6)	5 (2.7)
Other^b^	4	1	3	8 (4.4)

^a^n=number of patients with the specified species isolated at baseline; % based on total number of patients in each group (N).

^b^ *C. guilliermondii* (n = 2), *C. fermentati, C. intermedia, C. kefyr, C. rugosa*, *C. utilis,* and *C. metapsilosis* (n = 1 each).

### Efficacy

#### Primary Endpoint—Overall Response at Day 14 (mITT)

Overall cure was achieved in 60.5% (46/76) of the RZF 400 mg group, 76.1% (35/46) of the RZF 400/200 mg group, and 67.2% (41/61) of the CAS group (Table 3). The number of indeterminates was clustered in the RZF 400 mg group (Supplementary Table 5). Rates of overall cure with these indeterminates excluded were 69.7% (46/66) in the RZF 400 mg group, 81.4% (35/43) in the RZF 400/200 mg group, and 70.7% (41/58) in the CAS group.

**Table 3.** Primary Efficacy Endpoint: Overall Response at Day 14 (Microbiological Intent-to-Treat [mITT] Population)—Part A, Part B, and Combined

Overall Response, n (%)	Rezafungin Once Weekly 400 mg	Rezafungin Once Weekly 400 mg/200 mg	Caspofungin Once Daily 70 mg/50 mg
		**Part A**	
	N = 33	N = 31	N = 28
Overall cure [95% CI^a^]	19 (57.6)	22 (71.0)	18 (64.3)
	[39.2–74.5]	[52.0–85.8]	[44.1–81.4]
Failure/indeterminate	14 (42.4)	9 (29.0)	10 (35.7)
Failure	8 (24.2)	6 (19.4)	8 (28.6)
Indeterminate	6 (18.2)	3 (9.7)	2 (7.1)
		**Part B**	
	N = 43	N = 15	N = 33
Overall cure [95% CI^a^]	27 (62.8)	13 (86.7)	23 (69.7)
	[46.7–77.0]	[59.5–98.3]	[51.3–84.4]
Failure/indeterminate	16 (37.2)	2 (13.3)	10 (30.3)
Failure	12 (27.9)	2 (13.3)	9 (27.3)
Indeterminate	4 (9.3)	0	1 (3.0)
	**Combined (Part A + Part B)**		
	N = 76	N = 46	N = 61
Overall cure [95% CI^a^]	46 (60.5)	35 (76.1)	41 (67.2)
	[48.6–71.6]	[61.2–87.4]	[54.0–78.7]
Failure/indeterminate	30 (39.5)	11 (23.9)	20 (32.8)
Failure	20 (26.3)	8 (17.4)	17 (27.9)
Indeterminate	10 (13.2)	3 (6.5)	3 (4.9)

Abbreviation: CI, confidence interval.

^a^Exact 2-sided 95% CIs determined using the Clopper-Pearson method.

#### Secondary Endpoints, Outcomes by Diagnosis, and Time to Negative Blood Culture (mITT)

Clinical cure as assessed by the Investigator at day 14 was achieved in 69.7% (53/76) of the RZF 400 mg group, 80.4% (37/46) of the RZF 400/200 mg group, and 70.5% (43/61) of the CAS group. Additional secondary efficacy endpoints, including outcomes by diagnosis, are summarized in Table 4.

**Table 4.** Summary of Secondary Efficacy Endpoints by Diagnosis (Microbiological Intent-to-Treat [mITT] Population)—Parts A and B Combined

Efficacy Endpoint, n (%)	Rezafungin Once Weekly 400 mg	Rezafungin Once Weekly 400 mg/200 mg	Caspofungin Once Daily 70 mg/50 mg
By diagnosis			
Candidemia only	N = 57	N = 36	N = 48
Overall cure at day 14	35 (61.4)	25 (69.4)	31 (64.6)
By catheter status^a^			
Removed within 48 hours of candidemia diagnosis	12/17 (70.6)	8/10 (80.0)	9/17 (52.9)
Not removed	19/34 (55.9)	12/18 (66.7)	18/27 (66.7)
Mycological success			
Day 5	41 (71.9)	27 (75.0)	28 (58.3)
Day 14	38 (66.7)	25 (69.4)	32 (66.7)
PI assessment of clinical cure			
Day 14	41 (71.9)	27 (75.0)	34 (70.8)
Invasive candidiasis	N = 19	N = 10	N = 13
Overall cure at day 14	11 (57.9)	10 (100)	10 (76.9)
PI assessment of clinical cure			
Day 14	12 (63.2)	10 (100)	9 (69.2)

Abbreviation: PI, principal investigator.

^a^Patients with no central venous catheter are not listed.

**Figure 2.**
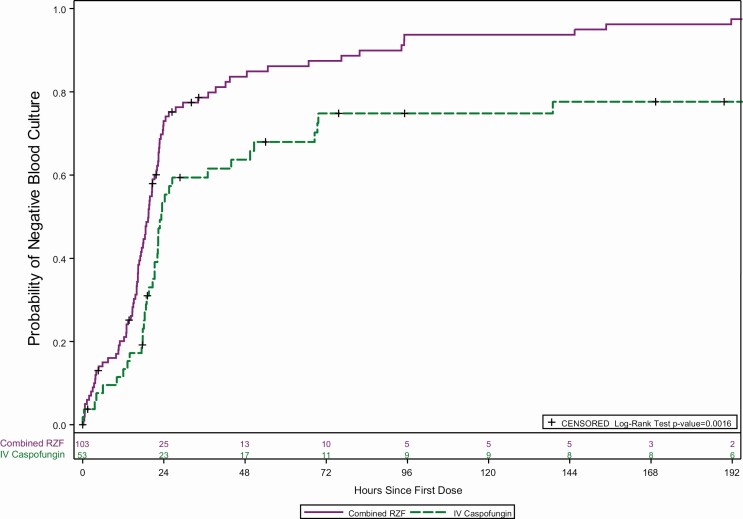
Time to negative blood culture following treatment with RZF versus caspofungin (*P* = .02; log-rank test, post hoc analysis). Abbreviations: IV, intravenous; RZF, rezafungin.

Evaluation of early outcomes, at day 5, for RZF (pooled) and CAS showed overall cure rates of 62.3% (76/122) and 55.7% (34/61), respectively, and similar results for mycological success (Table 5). Interestingly, the median time to a negative blood culture was 19.5 hours for RZF treated patients compared with 22.8 hours for CAS treated patients (ad hoc *P* = .02; Figure 2). The probability of a negative blood culture reached its maximum difference ~24 hours after the first dose.

**Table 5.** Secondary Efficacy Outcomes at Day 5 (Microbiological Intent-to-Treat [mITT] Population)—Parts A and B Combined

Endpoint at Day 5, n (%)	Rezafungin Once Weekly 400 mg N = 76	Rezafungin Once Weekly 400 mg/200 mg N = 46	Rezafungin Once Weekly Pooled N = 122	Caspofungin Once Daily 70 mg/50 mg N = 61
Overall cure	42 (55.3)	34 (73.9)	76 (62.3)	34 (55.7)
Mycological success	50 (65.8)	35 (76.1)	85 (69.7)	38 (62.3)

Day 30 ACM rates were 15.8% for the RZF 400 mg group, 4.4% for the RZF 400/200 mg group, and 13.1% for the CAS group.

Clinical cure rates at day 14 by baseline *Candida* species (*albicans* vs non-*albicans*, respectively) were 57.9% versus 80.5% in the RZF 400 mg group, 84.2% versus 81.3% in the RZF 400/200 mg group, and 73.5% versus 68.8% in the CAS group.

### Adverse Events

The percentage of patients who experienced at least 1 TEAE was 87.7% in the RZF 400 mg group, 92.5% in the RZF 400/200 mg group, and 80.9% in the CAS group. The most common TEAEs with an incidence of ≥5% for either drug were hypokalemia, diarrhea, and vomiting (Table 6). Severe TEAEs were reported in 35.8%, 32.1%, and 38.2% of patients in each group, respectively. Study drug-related TEAEs were reported in 8.6% of patients in the RZF 400 mg group, 11.3% of patients in the RZF 400/200 mg group, and 13.2% of patients in the CAS group.

**Table 6.** Treatment-emergent Adverse Events—Parts A and B Combined

System Organ Class Preferred Term, n (%)^a^	Rezafungin Once Weekly 400 mg N = 81	Rezafungin Once Weekly 400 mg/200 mg N = 53	Rezafungin Combined N = 134	Caspofungin Once Daily 70 mg/50 mg N = 68
≥1 TEAE	71 (87.7)	49 (92.5)	120 (89.6)	55 (80.9)
TEAEs with incidence of ≥5%^b^				
Hypokalemia	13 (16.0)	9 (17.0)	22 (16.4)	9 (13.2)
Diarrhea	7 (8.6)	11 (20.8)	18 (13.4)	10 (14.7)
Vomiting	6 (7.4)	8 (15.1)	14 (10.4)	5 (7.4)
Pyrexia	9 (11.1)	4 (7.5)	13 (9.7)	6 (8.8)
Anemia	6 (7.4)	7 (13.2)	13 (9.7)	4 (5.9)
Nausea	4 (4.9)	8 (15.1)	12 (9.0)	6 (8.8)
Pleural effusion	5 (6.2)	0	5 (3.7)	6 (8.8)
Abdominal pain	5 (6.2)	6 (11.3)	11 (8.2)	5 (7.4)
Septic shock	9 (11.1)	1 (1.9)	10 (7.5)	3 (4.4)
Constipation	3 (3.7)	3 (5.7)	6 (4.5)	5 (7.4)
Deep vein thrombosis	3 (3.7)	1 (1.9)	4 (3.0)	5 (7.4)
Dyspnea	1 (1.2)	1 (1.9)	2 (1.5)	5 (7.4)
Pneumonia	6 (7.4)	2 (3.8)	8 (6.0)	4 (5.9)
Hypotension	6 (7.4)	2 (3.8)	8 (6.0)	4 (5.9)
Insomnia	4 (4.9)	4 (7.5)	8 (6.0)	2 (2.9)
Peripheral edema	6 (7.4)	2 (3.8)	8 (6.0)	0
Sepsis	1 (1.2)	5 (9.4)	6 (4.5)	4 (5.9)
Cough	4 (4.9)	2 (3.8)	6 (4.5)	4 (5.9)
Bradycardia	2 (2.5)	2 (3.8)	4 (3.0)	4 (5.9)
Acute respiratory failure	2 (2.5)	1 (1.9)	3 (2.2)	4 (5.9)
Acute kidney injury	4 (4.9)	3 (5.7)	7 (5.2)	3 (4.4)
Decubitus ulcer	4 (4.9)	3 (5.7)	7 (5.2)	3 (4.4)

Abbreviation: TEAE, treatment-emergent adverse event.

^a^Patients who experienced multiple TEAEs were only counted once per preferred term.

^b^Based on reported incidence in either the rezafungin combined group or the caspofungin group.

Serious TEAEs (SAEs) were reported in 43.2%, 52.8%, and 42.6% of patients in the RZF 400 mg group, RZF 400/200 mg group, and CAS group, respectively (Supplementary Table 6). Patients with drug-related SAEs were reported in 1.2% of the RZF 400 mg group, 1.9% of the RZF 400/200 mg group, and 2.9% in the CAS group.

## DISCUSSION

In this trial, we compared the safety and efficacy of 2 treatment regimens of RZF once weekly to CAS once daily in the treatment of candidemia and/or invasive candidiasis. Not surprisingly, in this double-blinded trial, safety and tolerability between groups were comparable in that the most common AEs (hypokalemia, diarrhea, vomiting, and fever) were neither severe nor unexpected for this patient population [17]. As shown in Table 6, isolated differences in the incidence of individual TEAEs were observed between groups and treatments (eg, diarrhea, peripheral edema) but did not demonstrate a trend or pattern of concern. The safety findings of STRIVE, in the more than 200 patients enrolled, further validate the well-recognized safety profile of the echinocandin class of drugs and underscore the safety of RZF and its once-weekly dosing regimen. The ongoing phase 3 treatment trial of rezafungin 400/200 mg once weekly (ReSTORE; NCT03667690) will further contribute to the safety database on rezafungin.

The efficacy results of CAS treatment were consistent with previous findings, demonstrating the sensitivity and validity of STRIVE. Similarly, RZF outcomes were in line with expectations inferred from published trials of the 3 currently approved echinocandins. For the primary endpoint, the RZF 400/200 mg group had a higher rate of overall cure at day 14 compared with other groups. For the secondary endpoint of ACM at day 30, the lowest rate observed was in the RZF 400/200 mg group. The current trial, however, was not powered to evaluate differences in efficacy, and the moderate sample size limits interpretation of numerical differences between groups. Nevertheless, certain observations garnered interest and warrant discussion.

Much of the apparent difference in efficacy outcomes between the 2 RZF dose groups in STRIVE can be accounted for by the cluster of indeterminates in the RZF 400 mg group. True cure rates with indeterminate responses excluded, such as for the primary efficacy endpoint of overall response, were 81.4% for RZF 400/200 mg, 69.7% for rezafungin 400 mg, and 70.7% for CAS. The absence of toxicity or intolerability among the reasons for failure indicate that apparent differences in efficacy were not related to safety. The possibility of diminished efficacy due to paradoxical growth with higher echinocandin concentrations is likely to be raised but is unlikely to account for the observed differences in outcomes of the 2 RZF arms [18, 19]. While there has been substantial effort and investigation into this in vitro phenomenon over the past 2 decades, recently conducted clinical trials do not support this hypothesis [17, 20–23]. Furthermore, in the present trial, apparent differences between the RZF groups were already observed at day 5 (Table 5) when all RZF-treated patients had only received the same, initial 400-mg dose.

Prior clinical trials investigating echinocandins in the treatment of candidemia and IC have demonstrated consistently more favorable outcomes when compared to triazoles [2, 3, 24], and no significant differences between echinocandins has been shown upon direct comparison [17]. Since the initial landmark study demonstrating favorability of anidulafungin over fluconazole [24], the dosing strategies currently employed for echinocandins have been further explored and refined. A recent population pharmacokinetic study of micafungin in intensive care unit (ICU) patients found low serum drug concentrations; moreover, this study also found that standard dosing regimens are associated with a very low probability of attaining the target AUC:MIC value [25]. Additional work has similarly suggested that echinocandin peak concentration:MIC ratios correlate with efficacy, and higher peak concentration:MIC ratios have been shown to exhibit improved killing of *C. albicans* in animal models [26]. Lakota and colleagues demonstrated that front-loading of RZF (higher initial concentrations) results in greater fungicidal activity than more fractionated regimens [9]. Our observation of more rapid clearance of candidemia in the RZF-treated patients compared to those treated with CAS, with differences apparent within 24 hours of initial therapy, is consistent with these prior observations. They also support the higher overall efficacy rate observed in the RZF-treated groups at day 5 when compared to the rates seen in the CAS treatment arm in this trial (RZF pooled, 62.3% vs CAS, 55.7%). The rationally designed, front-loaded dosing strategy and unique pharmacokinetic properties of RZF result in prolonged therapeutic drug concentrations within peripheral tissues [27]. These findings may also support the mutant prevention concentration (MPC) concept and the potential to control resistance development by maintaining drug levels above the MPC for a period of time, as described by Zhao and colleagues [27]. Although this concept remains hypothetical, the potential implications are important for strategies to treat resistant and less-susceptible pathogens and underscore the clinical relevance of antimicrobial pharmacokinetics/pharmacodynamics [8, 26, 28, 29].

In conclusion, this phase 2, multicenter trial demonstrated safety, tolerability, and efficacy using once-weekly RZF in comparison with once-daily CAS followed by fluconazole, in the treatment of invasive candidiasis and candidemia. These data support the utility of a once-weekly dosing strategy with RZF for treatment of these infections and justify further evaluation in the ongoing randomized, phase 3 clinical trial (ReSTORE; NCT03667690) comparing once-weekly RZF 400/200 mg to CAS for the treatment of candidemia and invasive candidiasis.

## Supplementary Data

Supplementary materials are available at *Clinical Infectious Diseases* online. Consisting of data provided by the authors to benefit the reader, the posted materials are not copyedited and are the sole responsibility of the authors, so questions or comments should be addressed to the corresponding author.

ciaa1380_suppl_Supplementary_Material

## Notes

***Author contributions*.** All authors critically reviewed the manuscript and approved the final version for submission.

***Acknowledgments*.** This work was presented in part at Trends in Medical Mycology (TIMM) 2019, Nice, France. The authors thank the investigators, trial personnel, and patients who participated in the STRIVE trial. The STRIVE Trial Investigators include (listed alphabetically): George J. Alangaden (Henry Ford Health System); Svenja J. Albrecht (University of Mississippi Medical Center; Jackson Mycotic Research Center); Benito Almirante Gragera (University Hospital Vall d’Hebron); Mickael Aoun (Jules Bordet Institute); Ovidiu Horea Bedreag (Pius Brinzeu County Emergency Clinical Hospital); Steven Callens (University Hospital Ghent); Maria Angeles Ferre Colomer (University and Polytechnic Hospital La Fe); Georgios Daikos (LAIKO General Hospital); Cristina Chiutu Luminita (Craiova County Emergency Clinical Hospital); George Dimopoulos (University General Hospital Attikon); Jesus Fortun Abete (University Hospital Ramon y Cajal); Massimo Girardis (University Polyclinic Hospital of Modena); Manuela Aguilar Guisado (University Hospital Virgen del Rocio [HUVR]); Alexey Gritsan (Territorial Clinical Hospital); Shariq Haider (Hamilton Health Sciences); Maya Hites (Erasme Hospital); Luis E. Jauregui-Peredo (Mercy Health—St. Vincent Medical Center); Thomas Kerkering (Virginia Tech, Carilion School of Medicine); Daniel H. Kett (University of Miami Miller School of Medicine); Anastasia Kotanidou (General Hospital of Athens Evangelismos); Ana Loza (University Hospital of Valme); Francisco Marty (Harvard University, Brigham and Women’s Hospital Infectious Disease); Roberto Luzzati (Integrated University Health Authority of Trieste); Aleksei Mamonov (Mariinskaya City Hospital); Maria Merelli (Integrated University Hospital “Santa Maria della Misericordia” of Udine); Paloma Merino Amador (University Hospital Clinical San Carlos); Egidia Gabriela Miftode (Sf. Parascheva Clinical Hospital of Infectious Diseases); Jose Miguel Montejo Baranda (University Hospital Cruces); Minh-Hong Nguyen (University of Pittsburgh Medical Center); Vasileios Papastamopoulos (General Hospital of Athens Evangelismos); Patricia Munoz Garcia de Paredes (University General Hospital Gregorio Maranon); Michael Parry (Stamford Hospital); Debra Powell (Reading Hospital and Medical Center); John Pullman (Mercury Street Medical); Atanas Radinoff (University Multiprofile Hospital for Active Treatment); Juan Carlos Ramos Ramos (La Paz University Hospital); Sanjay Revankar (Harper University Hospital); Coleman Rotstein (University Health Network at University of Toronto); Agnes Sarkany (Fejer County St. Gyorgy University Teaching Hospital); Anne-Sophie Sauvage (University Hospital Center Sart-Tilman); Matthew D. Sims (Oakland University William Beaumont School of Medicine); Andrej Spec (Washington University School of Medicine); Dora Tancheva (University Multiprofile Hospital for Active Treatment and Emergency Medicine); Ellis H. Tobin (Albany Medical Center); Bernard Vandercam (Saint Luc University Hospital); Eric Van Wijngaerden (University Hospitals Leuven); Pierluigi Viale (Polyclinic S. Orsola-Malpighi); Donald C. Vinh (McGill University Health Centre—Research Institute); Igor Zabolotskikh and Tatiana Musaeva (Kuban State Medical University); Epaminodas Zakynthinos (University General Hospital of Larissa).

***Financial support.*** This work was supported by Cidara Therapeutics, Inc (San Diego, CA). Rezafungin is being developed by Cidara Therapeutics in partnership with MundiPharma (Cambridge, UK). Cidara Therapeutics was involved in the trial design, execution, and data analysis. Cidara Therapeutics and MundiPharma were involved in the reporting of the trial.

***Manuscript preparation*.** Christine Martinez and Tressa Chung (Scribant Medical) provided assistance in preparing and editing the manuscript with funding by Cidara Therapeutics.

***Potential conflicts of interest.*** G. R. T. has received grant support from Amplyx, Astellas, Cidara Therapeutics, Immy, Mayne, Scynexis, Viamet, and Wako and is a DRC member with Pfizer and received clinical trial support from F2G during the conduct of the study. A. Soriano has received grant support from Pfizer, personal fees from Merck, and honoraria as speaker from Pfizer, MSD, Angellini, Menarini, and Shionogi. J. V. received clinical trial support from Cidara Therapeutics outside the submitted work. P. M. H. has received a grant from Pfizer. J. H. has received a grant from MSD, has participated in activities as speaker for MSD, Pfizer, Astellas, and Zambon and has participated in advisory boards for MDS, Pfizer, Angelini, Menarini, and Zambon. M. B. has participated in advisory boards and/or received speaker honoraria from Achaogen, Angelini, Astellas, Bayer, Basilea, BioMérieux, Cidara Therapeutics, Gilead, Menarini, MSD, Nabriva, Paratek, Pfizer, Roche, Melinta, Shionogi, Tetraphase, VenatoRx, and Vifor and has received study grants from Angelini, Basilea, Astellas, Shionogi, Cidara Therapeutics, Melinta, Gilead, Pfizer, and MSD. L. O. has received grant support and /or consulting and speaking fees from Cidara Therapeutics, Scynexis, Amplyx, F2G, Merck, Astellas, Pfizer, Viracor, and Gilead. A. D. is a consultant to Cidara Therapeutics. R. M. V. is an employee and stockholder of Cidara Therapeutics. T. S. is an employee and shareholder of Cidara Therapeutics and has been issued a patent/copyright for rezafungin. P. G. P. has received grant support from Cidara Therapeutics, Amplyx, Scynexis, Mayne, IMMY, Astellas, and Merck. All other authors report no potential conflicts. All authors have submitted the ICMJE Form for Disclosure of Potential Conflicts of Interest. Conflicts that the editors consider relevant to the content of the manuscript have been disclosed.
