# Erratum to: Rezafungin Versus Caspofungin in a Phase 2, Randomized, Double-Blind Study for the Treatment of Candidemia and Invasive Candidiasis: The STRIVE Trial

**DOI:** 10.1093/cid/ciab402

**Published:** 2021-06-24

An error appeared in this article [Thompson GR III, Soriano A, Skoutelis A, et al. Rezafungin versus Caspofungin in a Phase 2, Randomized, Double-Blind Study for the Treatment of Candidemia and Invasive Candidiasis: The STRIVE Trial. Clin Infect Dis ciaa1380, https://doi.org/10.1093/cid/ciaa1380].

The figure of time to negative blood culture (Figure 2 of the article) was based on an analysis found to have 2 programming flaws discovered during further analyses of time to negative blood culture. First, the analysis was to be of patients with positive blood cultures, but patients with invasive candidiasis (IC) were included. Second, while the first of two required negative cultures was used as the time of culture clearance, assessment of blood cultures may not have accounted for cultures that were collected from 2 separate blood draws from different locations at approximately the same time. In some cases, the first negative blood culture used for the analysis was within minutes of a positive culture collected at approximately the same time. When these issues were identified, the analysis was repeated with the following adjustments (see text and Figure 2 below).

**Figure 2.**
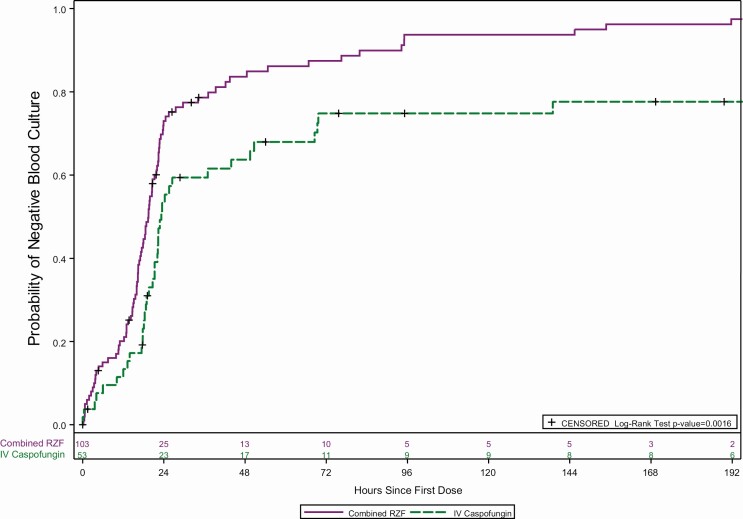
Time to negative blood culture following treatment with RZF versus caspofungin in patients with candidemia from the mITT population (*P* = .0016; log-rank test, post hoc analysis). Abbreviations: mITT, microbiological intent to treat; RZF, rezafungin. The first of two required negative cultures was used as the time of culture clearance, with the first negative blood culture drawn at least 1 hour after the last positive blood culture. The overall trend is unchanged, with significant differences between treatments observed as soon as ~24 hours after the first dose.

In the reanalysis, only patients with candidemia were included; patients with IC were excluded. Additionally, the programming parameters were updated to ensure that the first negative blood culture was drawn at least 1 hour after the last positive blood culture.

The reanalysis provides increased accuracy in the assessment of time to negative blood culture and, as shown in Figure 2 below, the overall conclusions are unchanged. The difference between treatments is statistically significant (P=.0016) and observed as early as ~24 hours after the first dose. Furthermore, the overall conclusions of the paper and the reported trial, re: the STRIVE Phase 2 trial of rezafungin treatment versus caspofungin, are unchanged. The trial sponsor apologizes for the error.

