# Supplementary material for: Rezafungin Versus Caspofungin in a Phase 2, Randomized, Double-blind Study for the Treatment of Candidemia and Invasive Candidiasis: The STRIVE Trial
Source: Clin Infect Dis. 2020 Sep 21;73(11):e3647–55. doi: 10.1093/cid/ciaa1380 (PMC8662762; doi:10.1093/cid/ciaa1380)
Supplement: ciaa1380_suppl_Supplementary_Material [file ciaa1380_suppl_supplementary_material.docx]

**SUPPLEMENTARY DATA**

**Supplementary Table 1. Countries of Enrollment (Alphabetical Order)**

| Belgium |
| --- |
| Bulgaria |
| Canada |
| Greece |
| Hungary |
| Italy |
| Romania |
| Russia |
| Spain |
| United States |

| **Supplementary Table 2. Demographics and Baseline Characteristics (ITT Population, Parts A and B)** | | | | | | | | | | | | |
| --- | --- | --- | --- | --- | --- | --- | --- | --- | --- | --- | --- | --- |
|  | | | Rezafungin once weekly  400 mg N=81 | | | Rezafungin once weekly  400 mg/200 mg N=57 | | | | Caspofungin once daily 70 mg/50 mg N=69 | | |
|  |  |  | Part A  N=35 | Part B  N=46 | | | Part A  N=36 | Part B  N=21 | Part A  n=36 | | | Part B  n=33 |
| Age in years | | |  | | | |  | |  | | | |
| Mean ± SD | | | 57 ± 16 | 61 ± 16 | | | 57 ± 14 | 65 ± 18 | 61 ± 17 | | | 58 ± 14 |
| Range | | | 24–88 | 25–87 | | | 26–84 | 24–91 | 24–93 | | | 25–85 |
| <65 years, n (%) | | | 24 (68.6) | 25 (54.3) | | | 24 (66.7) | 8 (38.1) | 17 (47.2) | | | 23 (69.7) |
| ≥65 years, n (%) | | | 11 (31.4) | 21 (45.7) | | | 12 (33.3) | 13 (61.9) | 19 (52.8) | | | 10 (30.3) |
| Sex, n (%) | | |  | | | |  | |  | | | |
| Male | | | 21 (60.0) | 23 (50.0) | | | 22 (61.1) | 14 (66.7) | 17 (47.2) | | | 21 (63.6) |
| Race, n (%) | | |  | | | |  | |  | | | |
| Asian | | | 0 | 0 | | | 1 (2.8) | 0 | 3 (8.3) | | | 0 |
| Black or African American | | | 5 (14.3) | 3 (6.5) | | | 5 (13.9) | 2 (9.5) | 2 (5.6) | | | 2 (6.1) |
| White | | | 30 (85.7) | 39 (84.8) | | | 28 (77.8) | 16 (76.2) | 31 (86.1) | | | 28 (84.8) |
| Other | | | 0 | 4 (8.7) | | | 1 (2.8) | 1 (4.8) | 0 | | | 0 |
| Not reported | | | 0 | 0 | | | 1 (2.8) | 2 (9.5) | 0 | | | 3 (9.1) |
| Ethnicity, n (%) | | |  | | | |  | |  | | | |
| Hispanic/Latino | | | 1 (2.9) | 7 (15.2) | | | 7 (19.4) | 2 (9.5) | 3 (8.3) | | | 4 (12.1) |
| Not Hispanic/Latino | | | 34 (97.1) | 39 (84.8) | | | 27 (75.0) | 19 (90.5) | 33 (91.7) | | | 26 (78.8) |
| Not reported | | | 0 | 0 | | | 2 (5.6) | 0 | 0 | | | 3 (9.1) |
| Diagnosis, n (%) | | |  | | | |  | |  | | | |
| Candidemia | | | 32 (91.4) | 30 (65.2) | | | 31 (86.1) | 15 (71.4) | 33 (91.7) | | | 23 (69.7) |
| Invasive Candidiasis | | | 3 (8.6) | 16 (34.8) | | | 5 (13.9) | 6 (28.6) | 3 (8.3) | | | 10 (30.3) |
| BMI^a^, mean ± SD kg/m^2^ | | | 25.2 ± 4.87 | 28.1 ± 8.31 | | | 27.2 ± 9.62 | 26.0 ± 6.09 | 27.1 ± 5.07 | | | 26.1 ± 6.17 |
| APACHE II Category, n (%) | | | | | | | | | | | | |
| 0–9 | | | 12 (34.3) | 11 (23.9) | | | 9 (25.0) | 6 (28.6) | 9 (25.0) | | | 8 (24.2) |
| 10–19 | | | 16 (45.7) | 23 (50.0) | | | 18 (50.0) | 8 (38.1) | 21 (58.3) | | | 16 (48.5) |
| ≥20 | | | 6 (17.1) | 11 (23.9) | | | 8 (22.2) | 6 (28.6) | 3 (8.3) | | | 6 (18.2) |
| Not available | | | 1 (2.9) | 1 (2.2) | | | 1 (2.8) | 1 (4.8) | 3 (8.3) | | | 3 (9.1) |
| APACHE II Score | | | n=34 | n=45 | | | n=35 | n=20 | n=33 | | | n=30 |
| Mean ± SD | | | 12.3 ± 7.16 | 14.1 ± 7.08 | | | 13.9 ± 6.83 | 14.5 ± 6.68 | 13.6 ± 6.89 | | | 14.4 ± 8.01 |
| Range | | | 2–31 | 2–30 | | | 2–27 | 3–28 | 2–35 | | | 1–33 |
| APACHE II=Acute Physiology and Chronic Health Evaluation II; BMI=Body Mass Index; SD=standard deviation.  ^a^BMI calculated by dividing weight (kg) by height (m^2^), based on patients with available data. | | | | | | | | | | | | |
| **Supplementary Table 3. Timing of Switch to Oral Step-Down Therapy (Safety Population)** | | | | | | | | | | | | |
| Timing of Switch to  Oral Step-Down, n (%) | Rezafungin once weekly 400 mg  N=81 | | | Rezafungin once weekly 400 mg/200 mg  N=53 | | | | | | Caspofungin once daily 70 mg/50 mg  N=68 | | |
| Study Day |  | | |  | | | | | |  | | |
| 4–6 | 11 (13.6) | | | 7 (13.2) | | | | | | 10 (14.7) | | |
| 7–9 | 6 (7.4) | | | 4 (7.5) | | | | | | 6 (8.8) | | |
| 10–12 | 2 (2.5) | | | 2 (3.8) | | | | | | 4 (5.9) | | |
| 13–15 | 1 (1.2) | | | 0 | | | | | | 1 (1.5) | | |
| 16–18 | 3 (3.7) | | | 2 (3.8) | | | | | | 2 (2.9) | | |
| 19–21 | 0 | | | 1 (1.9) | | | | | | 1 (1.5) | | |

| **Supplementary Table 4. Presence of Central Venous Catheters (mITT Population) – Parts A and B Combined** | | | |
| --- | --- | --- | --- |
| Catheter Type, n (%) | Rezafungin  once weekly  400 mg  N=76 | Rezafungin  once weekly 400 mg /200 mg  N=46 | Caspofungin once daily 70 mg/50 mg  N=61 |
| Peripherally Inserted (PICC) | 25 (37.3) | 12 (33.3) | 18 (32.7) |
| Triple/Quadruple Lumen | 30 (44.8) | 19 (52.8) | 30 (54.5) |
| Tunneled | 5 (7.5) | 5 (13.9) | 4 (7.3) |
| Other Vascular Catheter | 36 (53.7) | 24 (66.7) | 32 (58.2) |
|  |  |  |  |

| **Supplementary Table 5. Reasons for Failure or Indeterminate Response Outcomes in Overall Response (mITT Population) – Parts A and B Combined** | | | |
| --- | --- | --- | --- |
| Outcome, n (%)  Reasons for Outcome | Rezafungin  once weekly  400 mg  N=76 | Rezafungin  once weekly 400 mg /200 mg  N=46 | Caspofungin once daily 70 mg/50 mg  N=61 |
| Failure | 20 (26.3) | 8 (17.4) | 17 (27.9) |
| Death | 7 (9.2) | 2 (4.3) | 4 (6.6) |
| Mycological failure^a^ | 12 (15.8) | 6 (13.0) | 13 (21.3) |
| Recurrence of attributable systemic signs | 2 (2.6) | 0 | 2 (3.3) |
| Indeterminate Response | 10 (13.2) | 3 (6.5) | 3 (4.9) |
| Inadequate number of mycological cultures | 7 (9.2) | 3 (6.5) | 2 (3.3) |
| Assessment of systemic signs not completed | 6 (7.9) | 2 (4.3) | 1 (1.6) |
| Attributable systemic signs not reported at baseline | 1 (1.3) | 0 | 0 |

^a^Includes patients who had a change in antifungal therapy.

| **Supplementary Table 6. Treatment-Emergent Serious Adverse Events – Parts A and B Combined** | | | | |
| --- | --- | --- | --- | --- |
| Preferred Term,  n (%)^a^ | Rezafungin  once weekly  400 mg  N=81 | Rezafungin  once weekly  400 mg/200 mg  N=53 | Rezafungin combined  N=134 | Caspofungin once daily  70 mg/50 mg  N=68 |
| Any SAE | 35 (43.2) | 28 (52.8) | 63 (47.0) | 29 (42.6) |
| Study drug–related SAE | 1 (1.2) | 1 (1.9) | 2 (1.5) | 2 (2.9) |
| SAEs with incidence of ≥2%^b^ | | | | |
| Septic shock | 9 (11.1) | 1 (1.9) | 10 (7.5) | 2 (2.9) |
| Acute respiratory failure | 1 (1.2) | 0 | 1 (0.7) | 3 (4.4) |
| Sepsis | 1 (1.2) | 2 (3.8) | 3 (2.2) | 2 (2.9) |
| Multiple organ dysfunction syndrome | 2 (2.5) | 0 | 2 (1.5) | 2 (2.9) |
| Respiratory failure | 2 (2.5) | 1 (1.9) | 3 (2.2) | 1 (1.5) |
| Gastrointestinal hemorrhage | 2 (2.5) | 1 (1.9) | 3 (2.2) | 0 |
| SAE=serious TEAE; TEAE=treatment-emergent adverse event.  ^a^Patients who experienced multiple TEAEs were only counted once per preferred term.  ^b^Based on reported incidence in either the rezafungin combined group or the caspofungin group. | | | | |
